# The Utilization of Bevacizumab in Patients with Advanced Ovarian Cancer: A Systematic Review of the Mechanisms and Effects

**DOI:** 10.3390/ijms23136911

**Published:** 2022-06-21

**Authors:** Chih-Lin Mao, Kok-Min Seow, Kuo-Hu Chen

**Affiliations:** 1 Department of Obstetrics and Gynecology, Taipei Tzu-Chi Hospital, The Buddhist Tzu-Chi Medical Foundation, Taipei 231, Taiwan; moslin0722@gmail.com; 2 Department of Obstetrics and Gynecology, Shin Kong Wu Ho-Su Memorial Hospital, Taipei 111, Taiwan; M002249@ms.skh.org.tw; 3 Department of Obstetrics and Gynecology, National Yang-Ming Chiao-Tung University, Taipei 112, Taiwan; 4 School of Medicine, Tzu-Chi University, Hualien 970, Taiwan

**Keywords:** ovarian cancer, targeted therapy, angiogenesis, bevacizumab

## Abstract

Most ovarian cancer cases are diagnosed at an advanced stage (III or IV), in which a primary debulking surgery combined with adjuvant systemic chemotherapy is the standard management. Since targeted therapy is less toxic to human cells than systemic chemotherapy, it has drawn much attention and become more popular. Angiogenesis is a critical process during the proliferation of ovarian cancer cells. Currently, many studies have put emphases on anti-angiogenetic medication, such as bevacizumab, the first and most investigated angiogenesis inhibitor that can exert anti-neoplastic effects. Bevacizumab is a recombinant humanized monoclonal antibody that has been approved for first-line maintenance treatment of advanced ovarian cancer. This review is a summary of current literature about the molecular mechanisms of actions, safety, and effects of bevacizumab for use in advanced epithelial ovarian cancer. Some common side effects of bevacizumab will be also discussed. As an inhibitor of angiogenesis, bevacizumab binds to circulating vascular endothelial growth factor (VEGF) and thereby inhibits the binding of VEGF to its receptors on the surface of endothelial cells. Neutralization of VEGF prevents neovascularization and leads to apoptosis of tumor endothelial cells and a decrease in interstitial fluid pressure within the tumors, which allows greater capacity for chemotherapeutic drugs to reach specific targeted sites. Grossly, bevacizumab has demonstrated some significant therapeutic benefits in many randomized trials in combination with the standard chemotherapy for advanced epithelial ovarian cancer. Based on the available evidence, a higher dosage and a longer duration of bevacizumab appear to achieve better therapeutic effects and progression-free survival. On the other hand, patients with more severe diseases or at a higher risk of progression seem to benefit more from bevacizumab use. However, many unknown aspects of bevacizumab, including detailed mechanisms of actions, effectiveness, and safety for the treatment of ovarian cancer, warrant further investigation.

## 1. Introduction

Ovarian cancer ranks fifth place in cancer deaths among women, contributing higher death figures than any other cancer types of the female reproductive system. It is responsible for 14,070 deaths and 22,240 new cases in the United States. Most ovarian cancer cases are diagnosed at an advanced stage (III or IV), in which the overall 5-year survival rate is merely 30% [1]. A primary debulking surgery combined with adjuvant systemic chemotherapy is the standard management for advanced cases. Patients who are considered to be special cases, at risk if undergoing surgery, or with more aggressive diseases would begin with neoadjuvant chemotherapy, followed by an interval debulking surgery and adjuvant chemotherapy [2,3].

Over the past decade, the treatment of ovarian cancer has focused on targeted therapy as well as systemic chemotherapy. Since targeted therapy is less toxic to human cells than systemic chemotherapy, it has drawn much attention and become more popular. Currently, many studies have put emphases on anti-angiogenetic medications. For example, bevacizumab is the first and most investigated angiogenesis inhibitor that can exert anti-neoplastic effects [4]. Bevacizumab is a recombinant humanized monoclonal antibody that was approved by the US Food and Drug Administration (FDA) on 8 May 2020 for use as part of combination therapy with olaparib, a PARP inhibitor for first-line maintenance treatment of homologous-recombination-deficiency (HRD)-positive advanced ovarian cancer [5].

With regard to tumor neoangiogenesis, new blood vessels are essential to supply nutrients in favor of cancer cell growth, and they also contribute to the growth of tumors. Based on the fact, bevacizumab can be attached to vascular endothelial growth factor (VEGF) protein, thus acting on its target to inhibit or offset the growth effect of tumor cells [6]. While research has not indicated sufficient evidence to prove the increase in patients’ longevity [7], the effectiveness of treatment seems to be more obvious when the use of bevacizumab is combined with chemotherapy [8]. Even with dose-dense chemotherapy, the adjuvant bevacizumab has acceptable toxicity [9]. A recent study has also shown that upfront hyperthermic intraperitoneal chemotherapy (HIPEC) combined with bevacizumab-containing adjuvant chemotherapy can improve the prognosis of epithelial ovarian cancer [10]. This phase II, single-arm study enrolled 40 patients affected by advanced ovarian cancer. After complete debulking surgeries, all patients underwent HIPEC with bevacizumab addiction. The results showed maintenance with bevacizumab was feasible in 33 patients (82.5%), and its withdrawal was necessary for 1 patient only (2.5%) due to G3 hypertension. The study has demonstrated that HIPEC can be safely introduced in the upfront therapy of advanced ovarian cancer, along with the usage of bevacizumab [10].

In the past, patients with the BRCA mutation or genomic instability were usually treated with bevacizumab [11]. However, a PARP inhibitor named olaparib, functioning to inhibit polyp ADP ribose polymerase, an enzyme involved in DNA repair, can also be incorporated in the process of maintenance treatment [12]. A randomized, double-blind, international phase III trial investigated the effect of combining maintenance with olaparib and bevacizumab in patients with newly diagnosed, advanced, high-grade ovarian cancers regardless of BRCA mutation status. The median PFS was 22.1 months in the combining group and 16.6 months in the solo group (hazard ratio of disease progression or death: 0.59; *p* < 0.001). Adverse events were consistent with the established safety profiles of olaparib and bevacizumab. In patients with advanced ovarian cancers, combining maintenance with bevacizumab and olaparib could provide a significant progression-free survival (PFS) benefit in ovarian cancer patients with/without a BRCA mutation [12]. To sum up, the progress in medicine and technique has provided weapons, such as bevacizumab and olaparib, for humans to fight with ovarian and breast cancers. This review aims to summarize the molecular mechanisms of actions, safety, and effects of bevacizumab for use in advanced epithelial ovarian cancer. Some common side effects of bevacizumab will be also discussed.

## 2. Methods: Selection of the Reference Articles from the Literature 

**Figure 1:**
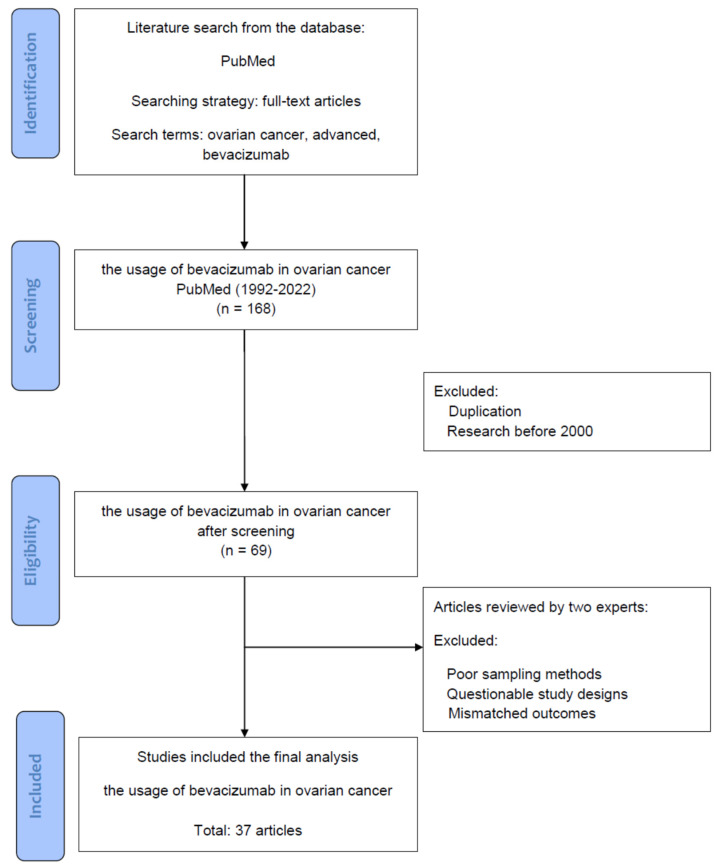
The flowchart of study selection (identification from the database, screening of the studies, selection of potential articles, and final inclusion) to identify eligible articles.

The literature was searched for basic and clinical studies reporting on the usage of bevacizumab in advanced ovarian cancer. Figure 1 illustrates the flowchart of identification, screening, selection, and inclusion of the references, which were retrieved from the literature. In this review, all of the articles we searched for references were retrieved from the database PubMed using the search terms “bevacizumab”, “advanced”, and “ovarian cancer” for the current topic. For further screening and selection, only full-text articles were considered to be included for a further analysis in the next stage. Our research team retrieved published articles from the literature in the database PubMed until 31 March 2022. Thus, the literature from 1 April 1992 to 31 March 2022 was searched as to identify potentially eligible articles for the review. In the second stage, both articles published before 2000 and duplicated articles were also excluded. After initial screening, two experts in the field individually inspected the contents of collected studies including demographics, research designs, and outcomes to identify eligible basic and clinical articles for exclusion and inclusion. In this stage, the studies with poor sampling methods, questionable research designs, or mismatched outcomes were excluded. The discrepancies between the experts were dealt with through direct discussion to reach a consensus. Using the search terms and strategies (identification from the database, screening of the studies, selection of potential articles, and final inclusion), all eligible studies were included in the review. From a total of 168 articles identified in the search, 37 articles finally met the criteria for inclusion.

## 3. Molecular and Cellular Mechanisms of Bevacizumab Actions

Angiogenesis is the formation of new blood vessels by remodeling and expansion of primary vessels. Normal angiogenesis is a highly ordered process under tight regulation, which ensures that developing or healing tissues receive an adequate supply of oxygen and nutrients [13]. Many positively and negatively acting factors influence angiogenesis, and among them the most well-characterized angiogenic factor is vascular endothelial growth factor (VEGF) [14]. VEGF belongs to a heparin-binding glycoprotein family consisting of six isoforms (VEGF-A through E and placental growth factor). VEGF-A shows prominent activity with vascular endothelial cells, primarily through its interactions with the VEGFR-1 and VEGFR-2 receptors found prominently on the membranes of vascular endothelial cells [15]. When VEGF-A binds to its receptor (VEGFR-2), the complex activates a signaling cascade mediated by mitogen-activated protein (MAP) kinase and PI3K/AKT/mTOR and results in the development of angiogenesis, increased vascular permeability, and lymphangiogenesis [6,16].

**Figure 2:**
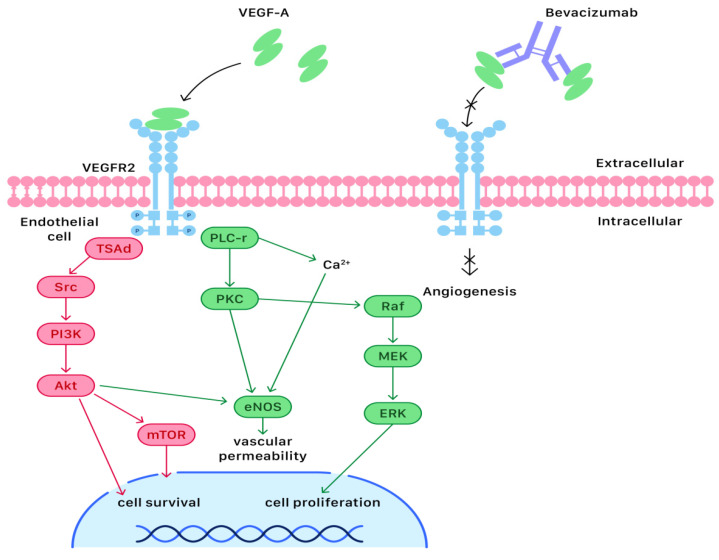
The molecular and cellular mechanisms of bevacizumab in angiogenesis of cancer cells.

Although VEGF-A binds VEGFR-1 with a higher affinity than it binds VEGFR-2, the biological effects of VEGF-A are thought to be mediated through VEGFR-2. Since VEGFR-2 is expressed on nearly all endothelial cells, the binding complex can exert widespread actions in the human body. During the process of VEGF-A binding, VEGFR-2 dimerizes and the tyrosine residues are phosphorylated, resulting in activation of the signal-transduction molecules phospholipase C-r (PLC-r), protein kinase C (PKC), consequent mitogen-activated protein kinase/ERK kinase (MEK), and extracellular signal-regulated kinase (ERK). On the other hand, T-cell-specific adapter (TSAd), Src, PI3K, Akt, and mTOR are also activated by the binding complex of VEGF-A and VEGFR-2 [17] (Figure 2).

In the first pathway, PLC-r stimulates the release of Ca^2+^ and activates PKC, which stimulates the Raf/MEK/ERK pathway and finally promotes cell proliferation. Ca^2+^ mobilization, PKC activation, and Akt activation are the key signaling events in VEGF-A-induced vascular permeability via endothelial nitric oxide synthase (eNOS) activity. In the second pathway, TSAd binds to phosphorylated tyrosine residues and then associates with Src kinases, which are expressed in endothelial cells, thus resulting in regulation of actin stress fiber and mediating VEGF-A-induced PI3K activation. PI3K activation further induces phosphorylation of Akt, thus inhibiting proapoptotic proteins and leading to cell survival [17]. The signaling cascades of both pathways, which are responsible for the development of angiogenesis, increased vascular permeability, and lymphangiogenesis, are illustrated in Figure 2.

Angiogenesis is a critical process during the proliferation of cancer cells. The production of VEGF is regulated by local oxygen concentration and usually overexpresses in malignant cells and tumors. Cancer cells often need a continuous supply of oxygen and nutrients, which means that these tissues with cancer cells are in a status of hypoxia. Hypoxia stimulates several complex pathways of cell signaling in cancer cells, including hypoxia inducible factor (HIF) cell signaling involved in tumor blood vessel formation and metastasis [18]. HIF binds to the hypoxia-response element present in the VEGF gene in a state of hypoxia and induces the transcription and translation of VEGF protein [19,20].

VEGF also enables endothelial and tumor cells to invade adjacent tissues by stimulating the endothelial cells to secrete proteolytic enzymes (e.g., plasmin, matrix metalloproteinases). These enzymes destroy the basement membranes of the precursor blood vessels and weaken the intracellular interactions in the vessel walls. Therefore, the original defense between normal and abnormal tissues is broken under such a condition. Unlike normal angiogenesis, which is highly ordered and strictly regulated, the angiogenesis of cancer cell is fenestrated, chaotic, and abnormal. This disorder creates blood vessels that are structurally and functionally abnormal and impairs the effective delivery of chemotherapeutic agents to the targeted cancer cells, which reduces the effects of chemotherapy [13].

The recurrence and metastasis of epithelial ovarian cancers are featured by the formation and invasion of abnormal tumor cells and vessels, accompanied by the drug resistance of therapeutic chemoagents. Bevacizumab is the first angiogenesis inhibitor to have been approved by the FDA. It is a humanized monoclonal immunoglobulin G antibody that is 93% human and 7% murine in protein sequence and inhibits all active isoforms of VEGF. As an inhibitor of angiogenesis, bevacizumab binds to circulating VEGF-A and thereby inhibits the binding of VEGF-A to its receptors on the surface of endothelial cells [21] (Figure 2). Neutralization of VEGF-A prevents neovasculature formation by limiting the blood supply and pruning of immature and abnormal blood vessels. These effects lead to apoptosis of tumor endothelial cells and a decrease in interstitial fluid pressure within the tumors, which allows greater capacity for chemotherapeutic drugs to reach specific targeted sites [22].

Angiogenesis is an ongoing process everywhere in the human tissues that need growth for biologic proliferation, establish transportation for fighting inflammation, and experience repair after sustaining an injury. Although angiogenesis can be divided into normal and abnormal vessel formation, the latter occurs in the majority of tumor tissues and is predominant in all kinds of angiogenesis in the human bodies. Thus, the neoangiogenesis inhibitor bevacizumab can act mainly in tumor tissues and be viewed as a targeted therapy for human cancers. Nevertheless, the inevitable effects of bevacizumab on ordinary vessel formation or normal tissues make its usage limited and account for its side effects in human bodies.

## 4. Therapeutic Effects of Bevacizumab

Table 1 is a summary of the clinical trials retrieved in the review, including trial name, phase of the study, response rate, progression-free survival, overall survival, and complete resection rate.

### 4.1. Data of the Phase II Trials

Three phase II trials regarding the usage of bevacizumab in advanced ovarian cancer were identified. They are listed as follows: OCTAVIA [23] and ANTHALYA [24] are international open-label, single-arm phase II studies. On the other hand, GEICO1205 [25] is an international, comparative, randomized phase II trial.

#### 4.1.1. OCTAVIA

The OCTAVIA study evaluated front-line bevacizumab plus weekly paclitaxel and q3w carboplatin. Most (74%) of the 189 treated patients had stage IIIC/IV disease. For the enrolled patients, the median progression-free survival (PFS) was 23.7 months, and the 1-year PFS rate was 85.6%. Most patients (90%) completed at least six cycles of chemotherapy. The OCTAVIA study revealed that the bevacizumab-containing regimen is active and tolerable [23].

#### 4.1.2. ANTHALYA

The Avastin Neoadjuvant THerapy in patients with Advanced ovarian cancer initialLY unresectAble (ANTHALYA) was also a multicenter, open-label, non-comparative phase II study. Ninety-five patients were randomized with a 2:1 ratio to undergo four cycles of neoadjuvant carboplatin and paclitaxel (CP) +/− three concomitant cycles of bevacizumab with a dosage of 15 mg/kg (BCP), followed by an interval debulking surgery (IDS). The study concluded that adding bevacizumab to neoadjuvant chemotherapy achieved an encouraging complete resection rate (CRR) with an IDS in patients with initially unresectable FIGO stage IIIC/IV ovarian, tubal, or peritoneal adenocarcinomas [24].

#### 4.1.3. GEICO1205

The GEICO1205 trial recruited 68 patients with newly diagnosed stage III/IV high-grade serous/endometrioid ovarian cancers. In the trial, the patients were randomized to receive four cycles of neoadjuvant chemotherapy with or without ≥three cycles of bevacizumab. The results of the trial revealed that in patients with initially unresectable ovarian cancer, the rate of complete macroscopic response with an interval debulking surgery was less than 10% in both treatment arms. Therefore, the trial concluded that adding bevacizumab to neoadjuvant chemotherapy did not improve the complete macroscopic response rate (primary end point). However, the surgical operability was improved [25].

### 4.2. Data of the Phase III Trials

The efficacy and safety data regarding the usage of bevacizumab are available from three randomized, double- blind, phase III trials of bevacizumab in advanced ovarian cancer: GOG218 [26], ICON7 [27,28] and ROSiA [29]. All of them focus on newly diagnosed advanced ovarian cancers.

#### 4.2.1. GOG218

In the GOG218 study, there were 1873 women with newly diagnosed stage III (incompletely resectable) or stage IV epithelial ovarian cancer who had undergone debulking surgeries to receive one of three treatments. All three included chemotherapies consisting of intravenous paclitaxel plus carboplatin for cycles 1 through 6, plus a study treatment for cycles 2 through 22, with each cycle having 3 weeks’ duration. The control treatment was chemotherapies with placebo added in cycles 2 through 22. The bevacizumab-initiation treatment was chemotherapies with bevacizumab added in cycles 2 through 6 and placebo added in cycles 7 through 22. Bevacizumab-throughout treatment was chemotherapy with bevacizumab added in cycles 2 through 22. The median PDS was 10.3, 11.2, and 14.1 months in the control group, the bevacizumab-initiation group, and the bevacizumab-throughout group, respectively. The median overall survival was 39.3, 38.7, and 39.7 months for the control group, the bevacizumab-initiation group, and the bevacizumab-throughout group, respectively. Although there is no significant difference in the overall survival rates, the full-course use of bevacizumab prolongs the median PFS about 4 months in patients with advanced epithelial ovarian cancers [26].

#### 4.2.2. ICON7

The ICON7 study enrolled 1528 women with ovarian cancers in one of two treatment regimens. Most of them (70%) had stage IIIC or IV ovarian cancers. The participants were randomly assigned in a 1:1 ratio to receive carboplatin and paclitaxel, given every 3 weeks for 6 cycles (the standard-chemotherapy group), or to the same regimen plus bevacizumab, given concurrently every 3 weeks for 5 or 6 cycles and continued for 12 additional cycles or until disease progression (the bevacizumab group). The median follow-up duration at the end of the trial was 48.9 months. No overall survival benefit of bevacizumab was recorded (restricted mean survival time 44.6 months in the standard chemotherapy group vs. 45.5 months in the bevacizumab group; log-rank *p* = 0.85). An updated analysis of the PFS showed no difference between these two treatment groups. However, further analyses of survival by stage, residual disease burden, and risk of recurrence revealed a benefit from bevacizumab with worsening prognostic factors. The overall survival in stage IV disease was 38.9 months in the bevacizumab group and 33.5 months in the control group. Similar patterns were also noted for the PFS (*p* = 0.014 for advanced stage, *p* = 0.005 for high risk patients). The results provide further evidence towards the optimum use of bevacizumab in the treatment of advanced ovarian cancer [27,28].

#### 4.2.3. ROSiA

The ROSiA study was a multinational prospective single-arm phase IIIB study, which recruited a patient population similar to ICON7. A total of 1021 patients from 35 countries had International Federation of Gynecology and Obstetrics stage IIB to IV or grade 3, stage I to IIA ovarian cancer. Unlike ICON7, prior neoadjuvant chemotherapy was permitted in the study. After debulking surgeries, patients received bevacizumab 15 mg/kg (or 7.5 mg/kg) every three weeks in combination with four to eight cycles of carboplatin and paclitaxel. Single-agent bevacizumab was continued until disease progression or up to 24 months (36 cycles) after chemotherapy discontinuation. The median duration of follow-up was 32 months. The overall median PFS was 25.5 months at the time of database lock. In the subgroup of 468 patients with advanced ovarian cancer (FIGO stage III and residual disease greater than 1cm, FIGO stage IV), median PFS was 18.3 months. The median PFS in the ROSiA study is the longest reported to date for frontline bevacizumab-containing therapy [29]. The result may raise the interesting hypothesis that the longer duration of bevacizumab contributes to an apparently extended PFS compared with the similar population within ICON7.

## 5. The Safety and Adverse Effects

The adverse effects of bevacizumab include hypertension, proteinuria, thromboembolism, neutropenia, infection, and gastrointestinal events. Most of the common adverse effects are listed in Table 2.

The most common grade 3/4 adverse events (AEs) of bevacizumab in the OCTAVIA study were hypertension (4.2%; 13.8% grade 2–4) and thromboembolic events (6.3%; 4.8% venous and 1.6% arterial). There was no proteinuria greater than grade 3 (grade 2 in 3.7% of patients). One patient (0.5%) experienced gastrointestinal perforation. No congestive heart failure nor reversible posterior leucoencephalopathy syndrome was reported. There were no treatment-related deaths [23].

In the ANTHALYA study, serious adverse events (SAEs) were more frequent in the neoadjuvant carboplatin–paclitaxel (CP) groups than the CP group with added bevacizumab (BCP). The most common treatment-emergent serious adverse events in the CP and BCP groups were gastrointestinal disorders (13% versus 7%, respectively), infections and infestations (8% versus 9%, respectively), and respiratory disorders (10% versus 2%, respectively). The addition of bevacizumab did not increase the rate of AEs or SAEs after neoadjuvant therapy compared with chemotherapy alone [24].

During the entire research period in the GEICO1205 study, AEs greater than grade 3 were significantly less common in the bevacizumab (CP + B) arm than the control (CP) arm (54% vs. 79%, respectively). AEs greater than grade 3 were significantly less common with bevacizumab-containing therapy than chemotherapy alone during neoadjuvant therapy (29% vs. 61%, respectively). In the 1-month period immediately following a major surgery, there was no significant difference in the incidence of AEs. The events included infection, hemorrhage, respiratory insufficiency, intestinal occlusion, ileus, lymphocele, and anemia [25].

In the GOG-0218 study, hypertension of grade 2 or greater was significantly (*p* < 0.001) more common with the bevacizumab than placebo, but this side effect led to discontinuation of bevacizumab in only 15 of the 608 patients (2.4%) in the bevacizumab-throughout group. There were no significant differences in the rates of other AEs, including gastrointestinal perforation or fistula, proteinuria of grade 3 or greater, neutropenia of grade 4 or greater or febrile neutropenia, venous or arterial thrombosis, and wound disruption. Fatal AEs were reported in 1.0%, 1.6%, and 2.3% of patients in the control group, bevacizumab-initiation group, and bevacizumab maintenance group, respectively. Most AEs were reported during the chemotherapy phase rather than the extended- therapy phase [26].

The ICON7 study reported that hypertension of grade 2 or greater occurred more frequently in the bevacizumab-containing arm compared with the chemotherapy-alone arm (18% vs. 2%, respectively). Bevacizumab was also associated with an increase in grade 1–2 mucocutaneous bleeding (36% vs. 7%, respectively), grade 3 or worse thromboembolic events (7% vs. 3%), and grade 3 or worse gastrointestinal perforations (ten [1%] vs. three [<1%]). In this study, five deaths related to treatment or disease per se were reported: one in the chemotherapy arm (0.1%, due to central nervous system ischemia) and four in the bevacizumab arm (0.5%, from gastrointestinal perforation, intracerebral hemorrhage, recurrent bowel perforation and ovarian cancer, and neutropenic sepsis and ovarian cancer, respectively) [27,28].

The most common all-grade AEs in the ROSiA study were hypertension (55%), neutropenia (49%), and alopecia (43%). The most common grade 3 or higher-grade AEs were neutropenia (27%) and hypertension (25%). Grade 3 proteinuria occurred in 4% of patients (no grade 4); 1.4% experienced grade 3 or higher-grade gastrointestinal perforation. The cumulative number of patients for whom these events occurred with time revealed the onset of hypertension and proteinuria. Hypertension occurred before month 6 of bevacizumab exposure in 77% of the patients, with a median time to onset of 2.1 months. Grade 3 or higher-grade hypertension occurred before month 6 of bevacizumab exposure in 158 of 252 (63%) patients [29].

However, the effects and side effects of bevacizumab remain to be investigated due to the relatively small sample size (<10,000/each) of the existent studies.

## 6. Results and Discussion

Angiogenesis is a critical process during the proliferation of cancer cells, and its progress is controlled by the key acting factor VEGF. The production of VEGF is regulated by local oxygen concentration and usually overexpresses in malignant cells and tumors. When VEGF-A binds to its receptor (VEGFR-2) on the membranes of vascular endothelial cells, the complex activates a signaling cascade mediated by mitogen-activated protein (MAP) kinase and PI3K/AKT/mTOR and results in the development of angiogenesis, increased vascular permeability, and lymphangiogenesis. VEGF also enables endothelial and tumor cells to invade adjacent tissues by stimulating the endothelial cells to secrete proteolytic enzymes such as plasmin and matrix metalloproteinases. These enzymes destroy the basement membranes of the precursor blood vessels and weaken the intracellular interactions in the vessel walls. Therefore, the original defense between normal and abnormal tissues is broken under such a condition.

The recurrence and metastasis of epithelial ovarian cancers are features of the formation and invasion of abnormal tumor cells and vessels, accompanied by the drug resistance of therapeutic chemoagents. As an inhibitor of angiogenesis, bevacizumab binds to circulating VEGF-A and thereby inhibits the binding of VEGF-A to its receptors on the surface of endothelial cells. These effects lead to apoptosis of tumor endothelial cells and a decrease in interstitial fluid pressure within the tumors, which allows greater capacity for chemotherapeutic drugs to reach specific targeted sites.

Abnormal vessel formation occurs in the majority of tumor tissues and is predominant in all kinds of angiogenesis. Thus, the neoangiogenesis inhibitor bevacizumab can act mainly in the tumor tissues and be viewed as a targeted therapy for human cancers. Nevertheless, the inevitable effects of bevacizumab on ordinary vessel formation or normal tissues make its usage limited and account for its side effects in human bodies. With similar or novel mechanisms, the debut of newer targeted-therapy drugs with possibly fewer adverse effects and better efficacy (more tissue-specific) is anticipated in the future.

Tracing back to the usage of anti-VEGF therapy, an earlier study was conducted to investigate the effects of chemotherapy–bevacizumab combination in newly diagnosed patients with stage ≥ IC epithelial Müllerian tumors, including ovarian cancers [30]. Patients received a combination of intravenous carboplatin, paclitaxel (175 mg/m^2^ IV), and bevacizumab (15 mg/kg IV) for six to eight cycles. Bevacizumab was omitted in the first cycle and continued as a single agent for 1 year. Among the 62 women enrolled in the phase II clinical trial, 45 women (73%) had ovarian cancers, 10 (16%) had peritoneal cancers, 4 (6%) had fallopian tube cancers, and 3 (5%) had uterine papillary serous tumors. The majority of patients (90%) had stage III or IV disease. Radiographic responses were documented in 21 (75%) of 28 women with measurable disease (11 complete responses and 10 partial responses), with CA-125 responses in 76% of patients (11 complete responses and 35 partial responses). The overall PFS rate at 36 months was 58%. The study revealed that chemotherapy with maintenance bevacizumab was feasible in the treatment of epithelial Müllerian tumors [30].

Grossly, bevacizumab has demonstrated some significant therapeutic benefits in many randomized phase II and phase III trials in combination with the standard chemotherapy for advanced epithelial ovarian cancer. This review could help clinicians to be aware of the recent evidence involving the usage of bevacizumab in advanced epithelial ovarian cancer, suggestive of future developments in this emerging area of investigation. A variety of research results exist in the present studies with respect to bevacizumab usage. Practically, some differences in results and conclusions among these studies in first-line treatment of ovarian cancer patients originate from the following: the eligibility criteria, drug dosage and duration of maintenance, and bevacizumab usage in neoadjuvant or adjuvant treatment.

Concerning the eligibility criteria, GOG218 only recruited III and IV staging patients, while in ICON7, the patients of ovarian cancers stage I-IIA (with clear cell histology and/or grading 3) and stage IIB were also included. Likewise, the patients in OCTAVIA and ROSiA studies were similar to the ICON7 population. In any case, both GOG218 and ICON7 trials revealed significantly improved progression-free survival (PFS) with the usage of bevacizumab added to standard chemotherapy. Several subgroup analyses classified according to stage and residual disease [31,32] revealed that patients with more advanced diseases or at a higher risk of progression may derive greater benefits from bevacizumab. In the GOG218 study, the usage of bevacizumab for patients with stage IV diseases showed better results in overall survival (OS) (40.6 months in the CPB arm vs. 32.8 in the CP arm; HR = 0.73). In the ICON7 study, the patients at a higher risk of progression showed a better median value of OS (30.3 months in the CP arm and 39.7 months in the CPB arm; HR = 0.64; *p* = 0.002) compared with those at a lower risk.

The dosage and maintenance duration in GOG218 was 15 mg/kg every 3 weeks for up to 15 months, and in ICON7, it was 7.5 mg/kg every 3 weeks for 12 months. Adding bevacizumab as a targeted therapy improved PFS more significantly in the GOG218 study than in the ICON7 study (3.8 months vs. 1.5 months). The lower dose and shorter maintenance in the ICON7 study may explain the results. The ROSiA study extended the usage of bevacizumab until progression or for up to 24 months, and the median PFS was 25.5 months (95% confidence interval: 23.7–27.6 months). In contrast, the median PFS was 14.1 months in the GOG218 study, 18.1 months in the ICON7 study, and 23.7 months in the OCTAVIA study. Nonetheless, extended bevacizumab usage in the ROSiA study increased the incidence of proteinuria and hypertension compared with previous trials. The results are compatible with the realistic clinical condition as described in a retrospective population study, which revealed that bevacizumab dosage showed cumulative toxicity and plateau effects on hypertension and proteinuria [33]. Close monitoring and effective management are very important for more extensive and safer use of bevacizumab in clinical situations, because proteinuria and hypertension are vital risk factors for renal and cardiovascular events [34].

Another trial evaluated the impact of intraperitoneal (IP) chemotherapy regimens on PFS among women with newly diagnosed advanced ovarian cancers [35]. Eligible patients (n = 1560) were randomly assigned to the total IV (IV paclitaxel and IV carboplatin), mixed IP 1 *(*IV paclitaxel and IP carboplatin), and mixed IP 2 (IV paclitaxel, IP cisplatin, and IP paclitaxel) groups. All participants received bevacizumab 15 mg/kg IV every 3 weeks in cycles 2 to 22. The median PFS duration was 24.9 months in the total IV, 27.4 months in the mixed IP 1, and 26.2 months in mixed IP 2 groups. The median overall survival for all enrolled patients was 75.5, 78.9, and 72.9 months, respectively. Compared with that in the total IV carboplatin and paclitaxel group, the duration of PFS was not significantly increased in either IP group when combined with bevacizumab [35]. This conclusion is contrary to the aforementioned study [10] conducted by Paris et al. More investigations are needed to explore the effect of chemotherapy routes when using bevacizumab for maintenance.

Based on the available evidence, a higher dosage and a longer duration of bevacizumab have achieved better therapeutic effects and PFS. On the other hand, patients with more severe diseases or at a higher risk of progression can benefit more from bevacizumab use. Possible explanations for the observed results are that an increased amount and duration of bevacizumab usage may exert a stronger action of anti-neovascular formation to inhibit tumor growth and that patients with more severe diseases or at a higher risk of progression usually have rapid cell proliferation and division, which appear more sensitive to anti-neovascularization agents and are subject to anti-tumor targeted therapy. Nevertheless, these postulates remain to be confirmed.

Anti-VEGF therapy, such as the usage of bevacizumab, can benefit against several cancer types, but drug resistance during the treatment may limit its therapeutic response. One possible mechanism for anti-VEGF drug resistance lies in the autocrine IL6/JAK/STAT3 signaling pathway, which is aberrantly hyperactivated in patients with chronic inflammatory conditions and in those with solid tumors or hematopoietic malignancies [36]. Multiple cell types in the tumor microenvironment produce IL-6, leading to activation of JAK/STAT3 signaling in both tumor cells and tumor-infiltrating immune cells, which can promote tumor-cell proliferation, survival, invasion, and metastasis. STAT3 is hyperactivated in tumor-infiltrating immune cells and acts to negatively regulate neutrophils, natural killer cells, effector T cells, and dendritic cells while positively regulating populations of myeloid-derived suppressor cells and regulatory T cells [36]. A clinical study has demonstrated that activation of the IL6/JAK/STAT3 pathway in tumor cells may provide a survival advantage of tumors during anti-VEGF treatment [37]. Furthermore, the data obtained from the treatment of ovarian cancers have shown poorer survival in patients with high levels of circulating IL6 [37]. Targeting components of the IL-6/JAK/STAT3 signaling pathway can inhibit tumor cell growth and relieve immunosuppression in the tumor microenvironment, and as of the present, inhibitors of IL-6, the IL-6 receptor, or JAKs have all received FDA approval for various malignancies. These provide a clinical implication for the combination use of bevacizumab and inhibitors of IL-6/JAK for decreasing anti-VEGF drug resistance and enhancing therapeutic response in the future.

The molecular level and pathologic mechanism of epithelial ovarian cancer and its treatment remain not fully understood. More investigations are required to explore the roles of anti-neovascularization targeted therapy in ovarian cancer. To minimize the heterogeneity of future research, standardization of several critical factors in ovarian cancer and associated treatment should be taken into consideration. Two of the important factors are the dosage and duration of targeted therapy, which have a significant impact on the therapeutic effects. Moreover, the severity and outcome in the patients with ovarian cancers also need standardization. Furthermore, a larger sample size is required to obtain reliable conclusions and to improve the reproducibility of the study results.

## 7. Conclusions

Angiogenesis is a critical process during the proliferation of ovarian cancer cells. As an inhibitor of angiogenesis, bevacizumab binds to circulating VEGF and thereby inhibits the binding of VEGF to its receptors on the surface of endothelial cells. Neutralization of VEGF prevents neovascularization and leads to apoptosis of tumor endothelial cells and a decrease in interstitial fluid pressure within the tumors, which allows greater capacity for chemotherapeutic drugs to reach specific targeted sites.

Grossly, bevacizumab has demonstrated some significant therapeutic benefits in many randomized trials in combination with the standard chemotherapy for advanced epithelial ovarian cancer. Based on the available evidence, a higher dosage and a longer duration of bevacizumab appear to achieve better therapeutic effects and progression-free survival. On the other hand, patients with more severe diseases or at a higher risk of progression seem to benefit more from bevacizumab use. However, many unknown aspects of bevacizumab, including detailed mechanisms of actions, effectiveness, and safety for the treatment of ovarian cancer, warrant further investigation.

## Figures and Tables

**Table 1:** A summary of the clinical trials retrieved in the review.

Clinical Trials	Phase of the Study	Arms	Number of Patients	Response Rate	Progression-Free Survival	Overall Survial (1 Year)	Overall Survial 2 Year)	Overall Survial	Complete Resection Rate
**OCTAVIA**	2	Single-arm	189	84.6%	23.7 months (90% CI: 19.9–26.4 months)	97.8%	92.1%		
**ANTHALYA**	2	CP vs. BCP	95	100%	-	-	-		58.6% vs. 51.4%
**GEICO1205**	2	CP vs. CP+B	68	100%	20.1 vs. 20.4	68 vs. 88	26 vs. 35		
**GOG218**	3	CP vs. CPB vs. CPB+B	1873	100%	10.3 vs. 11.2 vs. 14.1			39.3 vs. 38.7 vs. 39.7	
**ICON7**	3	CP vs. CPB	1528	100%	22.4 vs. 24.1			28.8 vs. 36.6	
**ROSiA**	3	Single-arm	1021	89%	25.5 months (95% CI: 23.7–27.6 months)	94% (95% CI, 93–96%)	85% (95% CI, 83–87%)		

**Table 2:** Adverse effects reported in clinical trials (%).

Clinical Trials (Regimen)	OCTAVIA	ANTHALYA		GEICO1205		GOG218			ICON7		ROSiA
		CP ^a^	BCP ^b^	CP ^c^	CP+B ^d^	CP ^e^	CPB ^f^	CPB+B ^g^	CP ^h^	CPB ^i^	
**Hypertension**	4.2			3	2.9	7.2	16.5	22.9	<1	6	55
**Proteinuria**	3.7					0.7	0.7	1.6	<1	1	4
**Fatigue/Asthenia**				9.1	2.9						
**Thromboembolic Events**	6.3			0	5.7	6.6	6.0	7.4	3	7	2.9
**Gastrointestinal Disorders**		13	7	3	5.7	1.2	2.8	2.6			
**Intestinal (Sub)occlusion**				12	5.7						
**GI Perforation**	0.5								<1	1	1.4
**Infections**		8	9	6.1	0						
**Respiratory Disorders**		10	2								
**Neutropenia**				6.1	11.4	57.7	63.3	63.3	15	17	49
**Thrombocytopenia**				3.9	2.9				2	3	
**Abdominal Pain**				6.1	0	41.6	41.5				
**CNS Bleeding**						0.8	1.3	2.1			

C: carboplatin; *p*: paclitaxel; B: bevacizumab, ^a^ Neoadjuvant treatment carboplatin AUC 5mg/mL/min + Paclitaxel 175 mg/m^2^, ^b^ Neoadjuvant treatment carboplatin AUC 5 mg/mL/min + Paclitaxel 175 mg/m^2^ + Bevacizumab 15mg/kg, ^c^ Neoadjuvant treatment carboplatin AUC 6mg/mL/min + Paclitaxel 175 mg/m^2^, ^d^ Neoadjuvant treatment carboplatin AUC 6 mg/mL/min + Paclitaxel 175 mg/m^2^ + Bevacizumab 15mg/kg, ^e^ Carboplatin AUC 6 mg/mL/min + Paclitaxel 175 mg/m^2^, ^f^ Carboplatin AUC 6mg/mL/min + Paclitaxel 175 mg/m^2^ + Bevacizumab 15 mg/kg(cycle 2 to 6), ^g^ Carboplatin AUC 6 mg/mL/min + Paclitaxel 175 mg/m^2^ + Bevacizumab 15 mg/kg(cycle 2 to 22), ^h^ Carboplatin AUC 5 or 6 mg/mL/min + Paclitaxel 175 mg/m^2^, ^i^ Carboplatin AUC 5 or 6 mg/mL/min + Paclitaxel 175 mg/m^2^ + Bevacizumab 7.5 mg/kg.
